# Comparison of the dose-response pharmacodynamic profiles of detemir and glargine in severely obese patients with type 2 diabetes: A single-blind, randomised cross-over trial

**DOI:** 10.1371/journal.pone.0202007

**Published:** 2018-08-16

**Authors:** Stefan Bilz, Miriam Flückiger, Fabian Meienberg, Claudine Falconnier, Ulrich Keller, Jardena J. Puder

**Affiliations:** 1 Division of Endocrinology and Diabetes, Kantonsspital St. Gallen, St. Gallen, Switzerland; 2 Division of Endocrinology, Diabetes and Metabolism, University Hospital Basel, Basel, Switzerland; 3 Division of Endocrinology, Diabetes and Metabolism, University Hospital Lausanne, Lausanne, Switzerland; Weill Cornell Medical College Qatar, QATAR

## Abstract

**Background:**

Despite their widespread use in this population, data on the pharmacodynamic (PD) properties of the insulin analogs detemir and glargine in severely obese patients with type 2 diabetes are lacking.

**Methods:**

The primary objective of the study was to compare the PD properties of two different doses of the basal insulin analogs detemir and glargine in patients with type 2 diabetes and a BMI > 35 kg/m^2^. PD data were derived from euglycemic clamp studies over 30 hours and each subject was studied for four times after the subcutaneous injection of a lower (0.8 U/kg body weight) and higher (1.6 U/kg body weight) dose of both detemir and glargine using a single-blind, randomised cross-over design.

**Results:**

Six male and four female patients with type 2 diabetes and a mean BMI of 43.2±5.1 kg/m^2^ (mean age 55.7±2 years, mean HbA1c 7.2±0.3%) completed the study. The total GIR_AUC0-30_ (mean difference 1224 mg/kg, 95%CI 810–1637, p = 0.00001), GIR_AUC0-24_ (mean difference 1040 mg/kg, 95%CI 657–1423; p = 0.00001), GIR_AUC24-30_ (mean difference 181 mg/kg, 95%CI 64–298; p = 0.004), GIR_max_ (mean difference 0.93 mg/kg/min, 95%CI 0.22–1.64, p = 0.01) and time to GIR_max_ (+1.9 hours, 95%CI 0.5–3.2; p = 0.009) were higher after the higher doses of both insulins, without significant differences between detemir and glargine. However, during the last 6 hours of the clamp the GIR_AUC24-30_ was significantly increased with glargine (mean difference 122 mg/kg, 95%CI 6–237, p = 0.043), reflecting a more pronounced late glucose lowering effect.

**Conclusions:**

A clear dose-response relationship can be demonstrated for both insulin analogs, even at very high doses in severely obese patients with type 2 diabetes. Compared to detemir, glargine has a more pronounced late glucose lowering effect 24–30 h after its injection.

**Trial registration:**

Controlled-Trials.com ISRCTN57547229.

## Introduction

Despite the recent introduction of novel antidiabetic agents such as incretins and SGLT2 inhibitors and the more widespread use of bariatric surgery insulin therapy is ultimately required to maintain glycemic targets in a large proportion of severely obese patients with type 2 diabetes. Current guidelines support the use of basal insulin if metformin or any dual or triple therapy fails to lower glucose values into the desired range [1,2]. The pharmacodynamic (PD) properties of the widely used basal insulin analogs detemir and glargine have been extensively studied in patients with type 1 and to a lesser extent in those with type 2 diabetes [3–16]. These studies report several important and potentially clinically relevant differences [17–19]. In patients with type 2 diabetes, different time-action profiles and distinct patterns regarding the suppression of endogenous insulin secretion and lipolysis have been reported [14]. Interestingly, a recent observation suggests that the degree of obesity may influence the PD effects of detemir [16]. The degree of obesity may also be an important determinant of subcutaneous insulin kinetics, and results obtained in overweight or moderately obese subjects may not be translated to those more severely obese [20,21]. These subtle but distinct individual properties may be well relevant for the choice of insulin, insulin dosing and the timing of basal insulin injections. Morbid obesity is frequently associated with severe insulin resistance thus requiring high basal insulin doses to suppress endogenous glucose production and lower fasting plasma glucose concentrations into the desired range [22]. However, only few data regarding the PD properties of detemir and glargine with no head-to-head comparison are available for these subjects. Therefore, the present study was designed to directly compare the PD properties of increasing doses insulin detemir and insulin glargine in severely obese (WHO grade 2 or higher) patients with type 2 diabetes over a 30 hour period in a randomised cross-over design.

## Subjects and methods

### Subjects

After final approval of the study protocol by the local ethical review board (Ethikkommission beider Basel, protocol 295/05, date of final approval Jan 16^th^ 2006) 10 patients with type 2 diabetes mellitus of at least 6 months duration, a HbA1c between 6.0 and 10.0% and a body mass index > 35 kg/m^2^ were recruited from the outpatients department of the Division of Endocrinology at the University Hospital Basel. Patients had to be treated with metformin, sulfonylureas or insulin and were in stable condition for the last three months. All patients provided written informed consent before enrolment, and all study procedures were conducted in accordance with the code of ethics of the World Medical Association (Declaration of Helsinki) for experiments involving humans. All study visits were performed between April 27^th^ 2006 and January 19^th^ 2007.

### Experimental design

A lower dose (0.8 Units/kg of body weight, LD) and a higher dose (1.6 Units/kg of body weight, HD) of both detemir and glargine were injected subcutaneously during four inpatient metabolic studies separated each by at least 2 weeks in random order. A random sequence of 4 numbers, each corresponding to one of the potential treatments was obtained for each patient using a web-based generator (www.random.org) by the investigators at the beginning of the first inpatient study to assign the sequence in which the four different treatments were applied. Patients, but not researchers, were blinded to the insulin types and doses. All antidiabetic medications, including basal insulins, were withheld at least four days prior to admission for the metabolic studies and glycemic control was achieved by multiple daily injections of a short acting insulin analog (lispro). After every study the subjects returned to their previous therapeutic regimen until four days before the next inpatient study. The euglycemic clamp technique was employed to derive PD data [23]. For each study, subjects were admitted to the metabolic ward of the University Hospital Basel at 7 a.m. after an overnight fast, and plasma glucose concentrations were lowered and kept stable at 5.5 mmol/l for at least one hour by an intravenous infusion of regular human insulin (Actrapid, NovoNordisk, Küsnacht, Switzerland). Thereafter, between 10 and 12 a.m., a single dose of detemir (Levemir, NovoNordisk, Küsnacht, Switzerland) or glargine (Lantus, Sanofi-Aventis, Meyrin, Switzerland) was subcutaneously injected into the anterior thigh. A maximum of 60 units of insulin was injected into a single site. Plasma glucose concentrations were measured every 10 to 30 minutes using a YSI glucose analyser and the glucose-oxidase method and clamped at 5.5 mmol/l for 30 h by frequently adjusting a 10% dextrose infusion. Additional blood samples were taken for the measurement of plasma C-peptide, glucagon, free fatty acids (FFA), detemir and glargine concentrations every two hours for the first six hours and every three hours thereafter. Patients remained fasting and in a supine or sitting position throughout the study period.

### Laboratory analyses

Plasma C-peptide (Adaltis, Italy) and glucagon (LINCO Research, Missouri, USA) were measured by a radioimmunoassay. Plasma FFAs were measured by an enzymatic test (Wako Chemicals GmbH, Neuss, Germany). Plasma glargine concentrations were measured by the LOCI (Luminescent Oxygen Channelling Immunoassay) technology with a lower detection limit of 8 pmol/l at NovoNordisk, Denmark [24]. Plasma detemir concentrations were measured using a specific enzyme-linked immunosorbent assay (ELISA; Capio Diagnostic) with a lower detection limit of 25 pmol/l as described previously [7].

### Statistical analysis

The primary endpoint of the study was the area under the curve of the glucose infusion rate (GIR_AUC_) required to maintain plasma glucose concentrations at 5.5 mmol/l during 30 hours after injection of the insulins and was calculated by the linear trapezoidal rule. To compare our data with other clamp studies that lasted 24 h, the first 24 h and the last 6 h were analyzed separately (GIR_AUC 0–24_ and GIR_AUC 24–30_). The secondary PD endpoints included the maximal glucose infusion rate (GIR_max_), the time to the maximal glucose infusion rate (tGIR_max_) and duration of insulin action. GIR_max_ and tGIR_max_ were estimated after fitting the individual GIR time curves using a polynomial function of 6^th^ order. Onset of action was defined as the time when the insulins were injected. The end of insulin action was defined as the time point when plasma glucose concentrations increased > 6.0 mmol/l after the glucose infusion had been stopped for at least 2 hours. The area under the curves were calculated using the trapezoidal rule. The PK endpoints included the plasma half-lives of both insulins. These individual plasma half-lives were calculated from the slope (k) of the decrease of plasma insulin concentrations after logarithmic transformation (T1/2 = ln2/k^-1^) assuming first order kinetics.

Differences between dose and type of insulin were calculated with the maximum-likelihood random-effects (MLE) model with insulin dose and type as fixed effects and subject as a random effect. Linearity between the effects of the two doses was assumed. Further baseline data (BMI, age, sex, diabetes duration, baseline HbA1c) were added to the model as random effects in a second step to test whether they modified the GIR response to insulin dose and type. The within patient variability of the GIR response to the two insulins was calculated according to the square root mean method [25,26]. Since plasma glucose concentrations remained below the threshold of 6.0 mmol/l after the glucose infusion had been stopped in a considerable number of experiments, the proportion of patients with an increase in plasma glucose concentrations > 6.0 mmol/l after the glucose infusion had been stopped and the proportion of patients requiring a continued glucose infusion at 24 and 30 hours were used as surrogates for the duration of insulin action and analyzed for different insulin doses and types using Fisher’s exact tests. The duration of the infusion and total dose of short acting insulin required to establish euglycemia at the beginning of the clamp studies were compared between the treatment groups using Kruskal Wallis tests. The PK data were analysed descriptively. Arithmetic means and standard errors, medians and range and mean differences (95% CI) between insulin dose and type are reported as appropriate and if not stated otherwise. The pharmacokinetic data are presented as harmonic means.

A previous study in patients with type 2 diabetes reported a dose dependent (0.4 U/kg body weight versus 1.4 U/kg body weight) increase of the GIR_AUC_ following the application of both glargine (571±647 to 2952±2028 mg/kg, mean±SD) and detemir (647±580 to 2171±1344 mg/kg). Using a two-tailed t-test for paired samples the sample size to detect a significant increase in the GIR_AUC_ between the lower and higher insulin doses in our study with a power of 80% and a type I error rate of 5% is 8 to 9. [11].

All calculations were made using XLSTAT Version 19.03 by Addinsoft, New York.

The study was retrospectively registered in the ISRCTN registry (https://doi.org/10.1186/ISRCTN57547229). However, patient recruitment did not start before the initial submission of the protocol to the registry and no changes to the protocol were made after this timepoint. Patient enrollment started before final registration for organizational reasons. The authors confirm that all ongoing and related trials for this intervention are registered.

## Results and discussion

### Baseline characteristics

**Fig 1:**
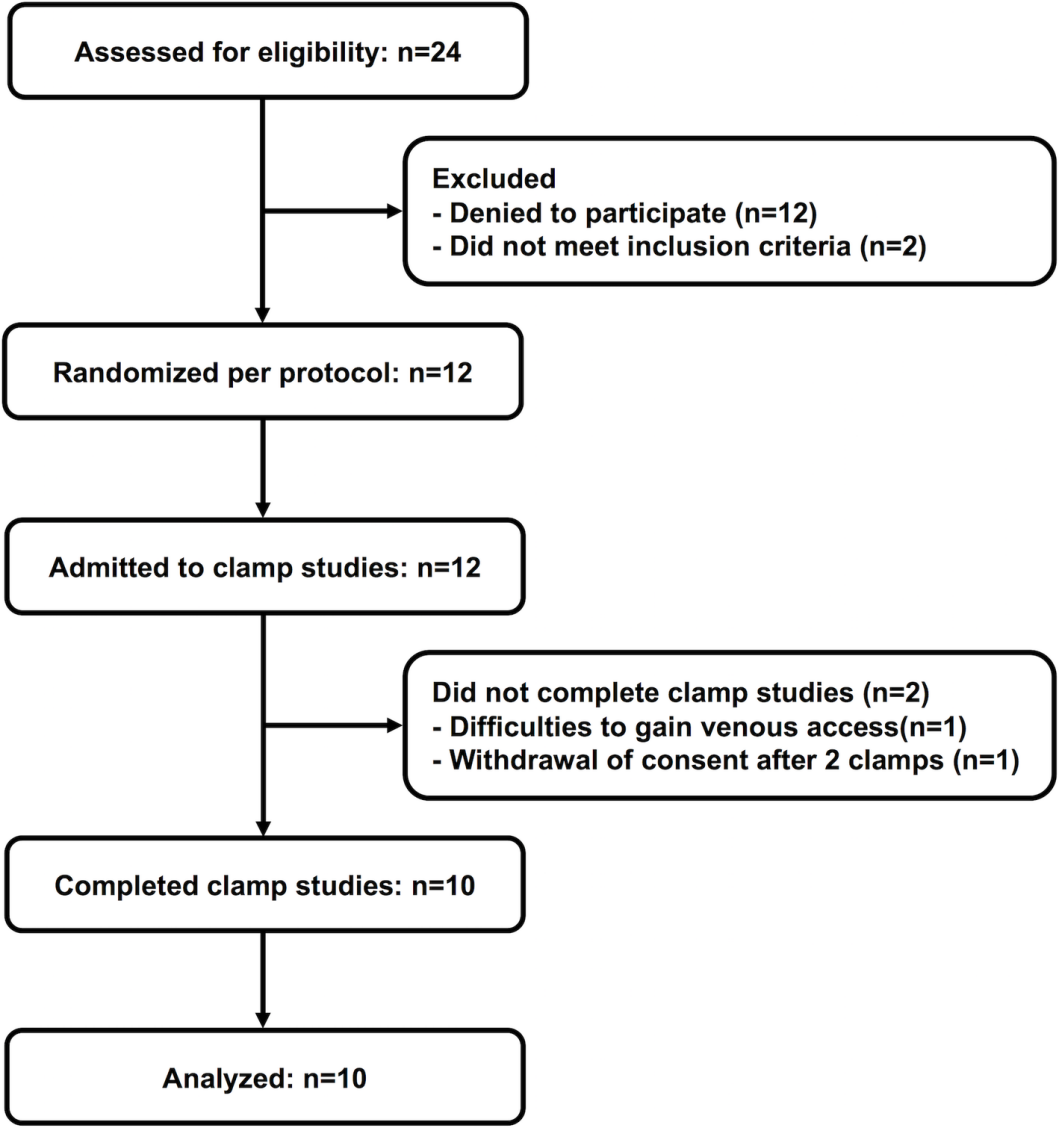
Patient flow.

Twelve patients were randomised to the study protocol. One subject terminated the study because of ongoing difficulties to gain venous access and one subject withdrew consent before completing the study (Fig 1). The baseline characteristics of patients completing the study are given in Table 1.

**Table 1:** Patient baseline characteristics.

Patient No.	Sex	Age *(years)*	Diabetes duration *(years)*	BMI *(kg/m*^*2*^*)*	HbA1c *(%)*	Oral antidiabetic therapy*	Basal insulin dose (U/kg BW*)
1	male	62	7	47.2	7.7	MET	0.60
2	female	57	6	42.6	7.8	MET, SU	none
3	female	61	38	50.0	6.5	none	0.40
4	male	39	5	52.2	7.7	MET	0.28
5	male	53	2	42.5	5.6	MET	none
6	female	51	8	40.0	7.6	MET	0.48
7	male	59	14	37.0	6.8	none	0.37
8	male	57	23	39.2	9.1	MET	0.54
9	male	61	9	38.0	7.6	MET	0.26
10	female	57	1	43.1	5.6	MET, SU	0.18
Median (range)		57 (39–62)	7.5 (1–38)	42.5 (37–52)	7.6 (5.6–9.1)		0.33 (0–0.60)

*MET =. Metformin; SU =. Sulfonylurea; U = Units, BW =. body weight

### Plasma glucose concentrations during the euglycemic clamp

**Fig 2:**
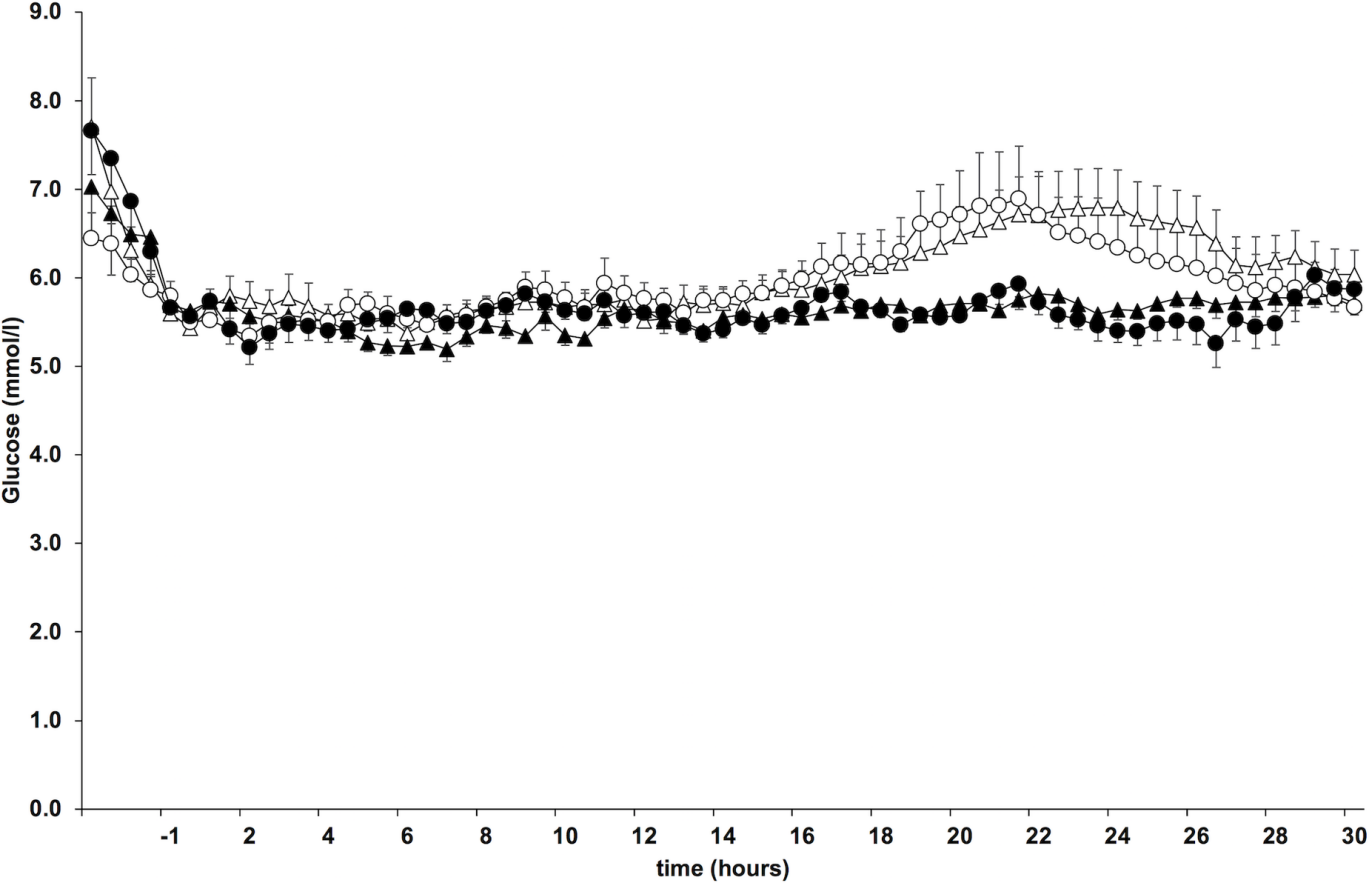
Plasma glucose concentrations. Mean (SE) plasma glucose during the 30 hours clamp period following the four different treatments (△ detemir lower dose, ▲ detemir higher dose, ○ glargine lower dose, ● glargine higher dose).

The dose and duration of short acting insulin infusion required to lower and maintain plasma glucose at 5.5 mmol/l at the beginning of the clamp studies was comparable, regardless of insulin dose and type (S1 Table). Plasma glucose concentrations remained stable at 5.5 mmol/l throughout the 30 h clamp period when the higher doses of both insulins were applied but increased temporarily after ~ 16 h during the LD studies and decreased again after ~ 22 h (Fig 2). This resulted in a significantly lower mean plasma glucose concentration during the last 12 hours when the higher insulin doses were given (-0.78 mmol/l, 95% CI -1.30 - -0.27; p = 0.005). The glucose over time curves appeared lower after glargine when compared to detemir during the last 6 hours of the LD studies, but this difference was not statistically significant (mean difference between detemir and glargine 0.34 mmol/l, 95% CI -1.18–0.49, p = 0.416).

### Pharmacodynamics—GIR_AUC_, GIR_max_ and tGIR_max_

**Fig 3:**
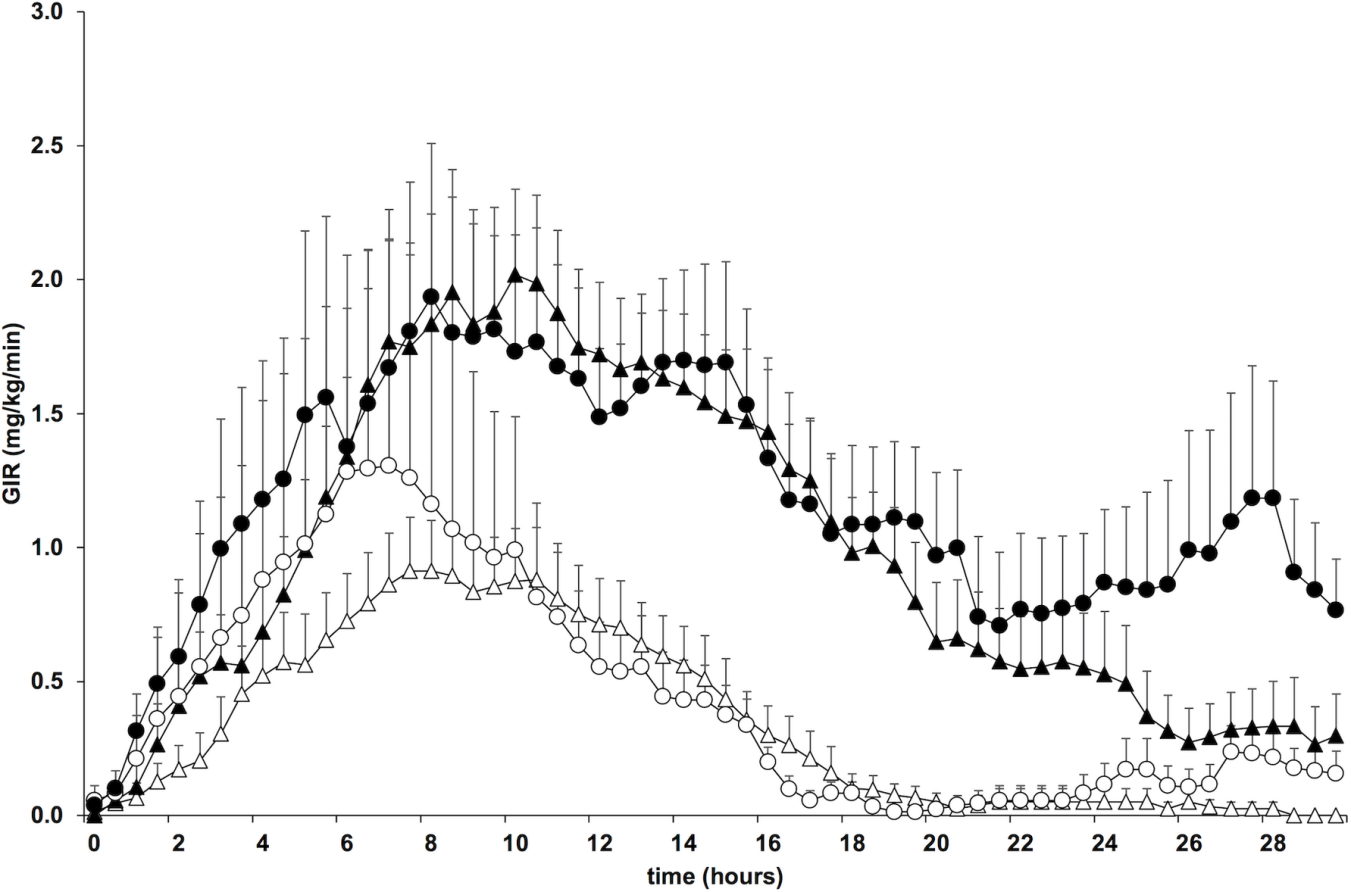
Glucose infusion rate. Mean (SE) glucose infusion rate during the 30 hours clamp period following the four different treatments (△ detemir lower dose, ▲ detemir higher dose, ○ glargine lower dose, ● glargine higher dose).

The pharmacodynamic responses to both insulins were clearly dose-dependent (Fig 3, Table 2). Compared to the LD studies, the total GIR_AUC 0–30_ (mean difference 1224 mg/kg, 95% CI 810–1637; p = 0.00001), GIR_AUC 0–24_ (mean difference 1040 mg/kg, 95% CI 657–1423; p = 0.00001) and GIR_AUC 24–30_ (mean difference 181 mg/kg, 95% CI 64–298; p = 0.004) were increased when the higher insulin doses were used. The GIR_AUC 0–30_ and GIR_AUC 0–24_ did not differ between the two insulins. During the last 6 h of the clamp, however, the GIR_AUC 24–30_ was significantly higher after glargine when compared to detemir (mean difference 122 mg/kg, 95% CI 6–237, p = 0.04), indicating a more pronounced late glucose lowering effect of glargine. The GIR_max_ was increased after the HD compared to the LD studies of both insulins (mean difference 0.93 mg/kg/min, 95% CI 0.22–1.64, p = 0.01) but was comparable between insulin types. The time to GIR_max_ was delayed when the higher insulin doses were applied (+1.9 hours, 95% CI 0.5–3.2; p = 0.009), but was also similar between insulin types (p = 0.45). The within patient variability was similar for both insulins for the total GIR_AUC_ and GIR_max_ (all p≥0.05), but significantly lower following detemir for the time to GIR_max_ (p = 0.011, S2 Table). This suggests that the time to peak action is more predictable following detemir. Addition of sex, age, BMI, diabetes duration and baseline HbA1c as random effects to the statistical model did not change the GIR responses (data not shown), suggesting that these parameters had no significant impact on the pharmacodynamic responses to the insulins in our study. The mean duration of insulin action could not be derived since a continued glucose infusion until the end of the study was necessary in a considerable portion (30%) of the studies. However, the percentage of patients requiring an ongoing glucose infusion to maintain target plasma glucose concentrations at 24 and 30 hours of the clamps was significantly higher during the HD experiments (HD vs. LD: 72% vs. 16% at 24 hours; p = 0.001, 50% vs. 10% at 30 hours, p = 0.01). At 30 but not at 24 hours significantly more patients required a continued glucose infusion when glargine was used (24 hours: glargine 50%, detemir 37%, p = 0.419; 30 hours: glargine 50%, detemir 11%, p = 0.01). Furthermore, the proportion of patients with an increase in the plasma glucose concentration above the threshold of 6.0 mmol/l at least 2 hours after the glucose infusion had been stopped was significantly higher during the lower dose studies (LD 79%, 95% CI 61–97; HD 33%, 95% CI 11–55, p = 0.008) and following the application of detemir (detemir 79%, 95% CI 61–97, glargine 33%, 95% CI 11–55, p = 0.008). Together, these data support a clear dose dependency of the duration of insulin action and suggest a more prolonged action of glargine when compared to detemir.

**Table 2:** Pharmacodynamic endpoints calculated from glucose infusion rates (GIR) during the clamp studies.

	Detemir		Glargine		*p-value*	
	*LD*	*HD*	*LD*	*HD*	*Insulin type*	*Insulin dose*
**GIR**_**AUC 0–30**_ (mg/kg)	608 (280–935)	1742 (944–2539)	784 (-133-1702)	2096 (906–3286)	*0*.*231*	*0*.*00001*
**GIR**_**AUC 0–24**_ (mg/kg)	598 (283–914)	1630 (922–2339)	729 (-193-1652)	1781 (787–2774)	*0*.*506*	*0*.*00001*
**GIR**_**AUC 24–30**_ (mg/kg)	9 (-5-24)	111 (-1-224)	55 (-1-112)	315 (36–595)	*0*.*043*	*0*.*004*
**GIR**_**max**_ (mg/kg/min)	0.93 (0.48–1.38)	1.99 (1.23–2.74)	1.28 (-0.48–3.04)	2.09 (0.84–3.34)	*0*.*564*	*0*.*014*
**Time to GIR**_**max**_ (hours)	9.8 (8.5–11.1)	11.1 (9.9–12.2)	9.7 (7.5–11.9)	12.2 (10.3–14.0)	*0*.*448*	*0*.*009*

GIR_AUC_ denotes area under the curve of the glucose infusion rate. GIR_max_ denotes maximal glucose infusion rate. LD denotes lower insulin dose, HD denotes higher insulin dose. Data are means and 95% CI. P-values are given for differences between insulin type and insulin dose.

### Plasma FFA, C-peptide and glucagon concentrations

**Fig 4:**
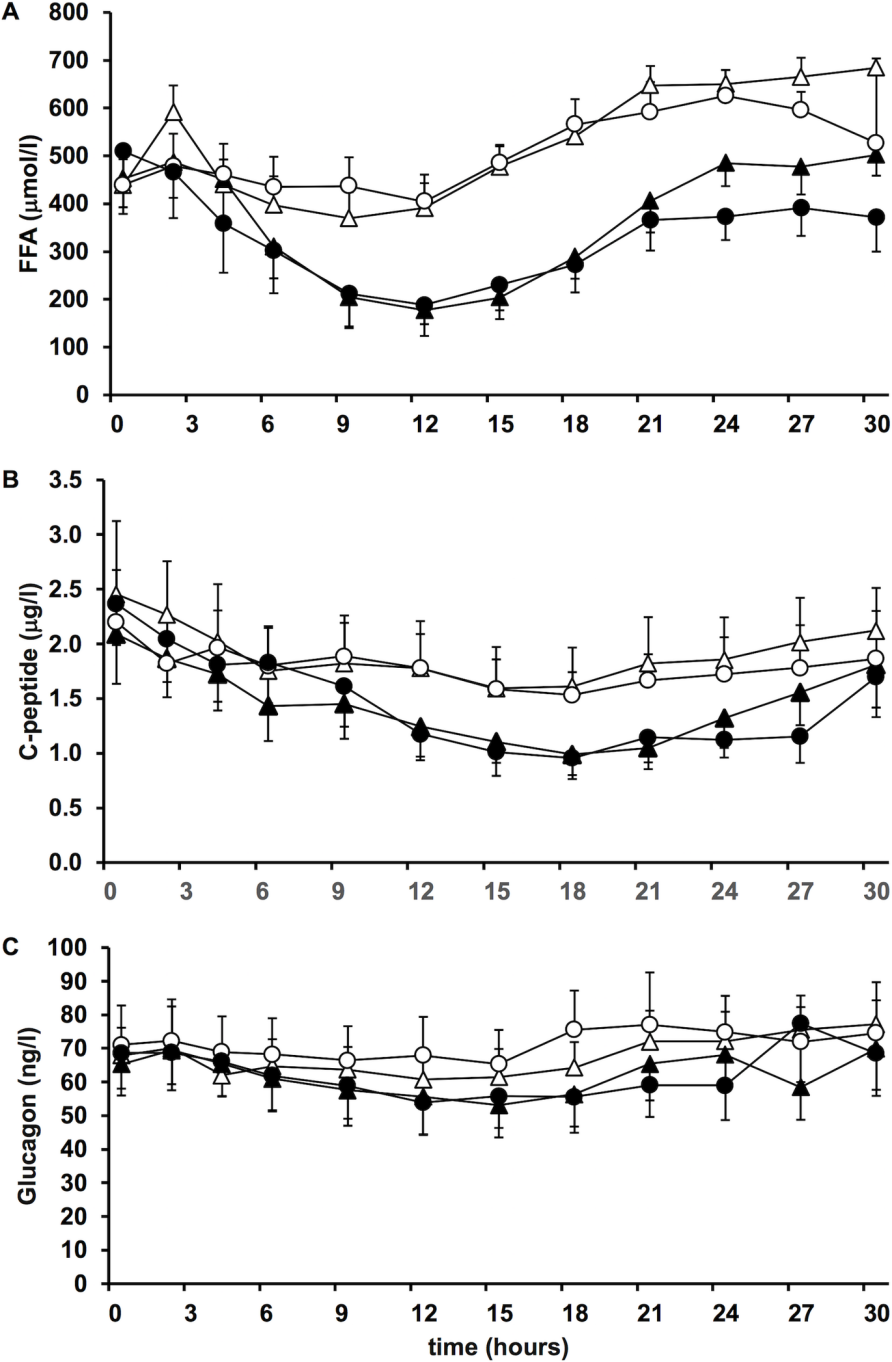
Plasma FFA, C-peptide and glucagon. Mean (SE) plasma concentrations of FFA (A), C-peptide (B) and glucagon (C) during the 30 hours clamp period following the four different treatments (△ detemir lowerdose, ▲ detemir higher dose, ○ glargine lower dose, ● glargine higher dose).

Plasma concentrations of FFA reached their nadir between 9 and 12 hours of the clamp and increased thereafter during all experiments (Fig 4A). Compared to the LD studies FFA were significantly decreased when the higher insulin doses were applied (mean difference 211 µmol/l, 95% CI 93–331; p = 0.001, S3 Table). No difference between the effects of detemir and glargine on plasma FFA concentrations was observed. Similarly, plasma C-peptide concentrations decreased when the high doses of both insulins were injected with a nadir occurring around 18 hours after the start of the studies (Fig 4B). The mean decrease in plasma C-peptide concentrations was significantly increased during the HD when compared to the LD studies (mean difference 0.32 μg/l, 95% CI 0.02–0.64; p = 0.04, S3 Table) but did not differ between insulin types. A non-significant trend towards lower glucagon concentrations was observed during the HD when compared to the LD studies (mean difference 5.1 ng/l, 95% CI -0.41–10.59, p = 0.07, Fig 4C, S3 Table). These effects of the higher insulin doses were well reflected when the area under the curves of plasma FFA-, C-peptide and glucagon concentrations were calculated (S4 Table).

### Pharmacokinetics

Plasma concentrations of both insulins remained well measurable until the end of the clamp at 30 hours (S1 Fig). At 30 hours of the clamp, plasma concentrations of glargine and detemir had decreased to 54 and 11% of their peak concentrations, respectively. The half-lives of insulin glargine and detemir were 40.7 and 16.2 hours, respectively, with no apparent major effect of the different insulin doses (detemir 16.0 and 16.3 hours, glargine 42.1 and 51.6 hours, LD and HD).

## Discussion

This single-center, randomized cross-over study explored for the first time the pharmacodynamic (PD) differences of the widely used basal insulin analogs detemir and glargine in WHO grade 2–3 obese individuals with type 2 diabetes. Since up-titration to high basal insulin doses is frequently necessary to control fasting glucose levels in severely obese, insulin resistant subjects relatively high (LD, 0.8 U/kg) and very high (HD, 1.6 U/kg) insulin doses were applied as single injections, and their effects were assessed during 30 hours thereafter. A clear dose-response relationship of their metabolic actions has been previously described for both detemir and glargine with doses up to 1.4 U/kg (detemir) and 2.0 U/kg (glargine) in less obese (WHO grade 1–2) patients [11,13,27]. Furthermore, studies performed in overweight or only mildly obese (WHO grade 1) type 2 diabetic patients described several distinct differences between detemir and glargine with regards to their metabolic properties [14–16]. The randomised cross-over design chosen allowed us to test both whether a dose-response relationship is still demonstrable if similarly high insulin doses are applied to very obese patients and if the metabolic actions of detemir and glargine are comparable in this population. According to our sample size calculation the number of participants was clearly sufficient to draw firm conclusions regarding the effect the different insulin doses applied on the GIR_AUC_.

Following s.c. administration of the higher insulin doses plasma glucose values remained stable at the target concentration throughout the 30 h clamp period. When the lower doses of the insulins were applied glucose concentrations increased slightly after ~16 hours of the clamp and returned towards the target concentration towards the end of the study period. This temporary increase is likely accounted for by a waning effect of insulin at this time. The subsequent decrease of plasma glucose may be well explained by the resumption of endogenous insulin secretion as well as prolonged fasting, as indicated by the time course of plasma C-peptide and FFA concentrations [28].

With regards to the primary endpoint, the total GIR_AUC_, a highly significant effect of the insulin dose but not the insulin type was observed. This confirms a clear dose-response relationship of the metabolic action for both insulins even if very high insulin doses are applied to severely obese individuals. The GIR-over-time curves demonstrate a flat but distinct peak effect of both detemir and glargine and the glucose lowering effect was well sustained for at least 24 hours with the higher doses of both insulins. The major pharmacodynamic difference between detemir and glargine was a more pronounced glucose lowering action of glargine during the last six hours of the 30 hours clamp period. Since plasma glucose concentrations did not increase above baseline in many clamps, mostly after the higher insulin doses had been applied, we cannot provide an accurate quantitative estimate of the duration of action of the insulins. However, the surrogate measures employed (number of patients with rising plasma glucose after cessation of glucose infusion) clearly point to a dose-dependent increase in the duration of action of both insulins and support the view of a more prolonged action of glargine compared to detemir. Together, these results are novel and have, to our best knowledge, not been previously reported in a comparable population. The PD results are further supported by the finding that the suppression of endogenous insulin secretion mirrored by plasma C-peptide concentrations and inhibition of lipolysis reflected by plasma free fatty acid concentrations by the two insulins were comparable but again clearly dose-dependent. Pharmacokinetic data of the two insulins are difficult to interpret since their plasma concentrations differ markedly due to the extensive albumin binding of detemir. Nevertheless, the pronounced late glucose-lowering effect of glargine when compared to detemir is supported by the slow decrease in its plasma concentrations which still were > 50% of the maximal levels at the end of the study. Therefore, our findings suggest that once daily dosing of both basal insulin analogs is well suitable in obese subjects with type 2 DM, and up-titration to very high doses may provide additional glucose lowering effects. Since both insulins at both doses discerned a clear and predictable peak effect, timing of the insulin injection may be critical despite the protracted action of both insulins, as recently suggested [14,18,29].

Several previous studies have reported on the PD effects of detemir and glargine in type 2 diabetic subjects and varying degrees of obesity with three head-to head comparisons. In agreement with our results Wang et al. demonstrated a clearly dose-dependent metabolic effect of a single injection of glargine (0.5 to 2.0 U/kg) throughout the 24 hours study period in less obese patients (mean BMI 36 kg/m^2^) with otherwise comparable characteristics [13]. A very similar PD profile of glargine with a flat peak and duration of action up to 24 hours had previously been described by Luzio et al. in insulin-naive type 2 diabetic subjects with a mean BMI of 31 kg/m^2^ [12]. The same authors compared the PD profiles of detemir and glargine in obese (mean BMI 31 kg/m^2^) subjects using a single injection, single-dose (0.5 U/kg), cross-over design and found also no difference in the GIR_AUC_ over 24 hours [15]. However, a significantly flatter profile and less between subject variation of insulin action was observed after glargine. Similarly, a linear and comparable dose-response effect (0.4, 0.8 and 1.4 U/kg) on the major pharmacodynamic parameters was reported for detemir and glargine over 24 hours in the single-injection, cross-over study of Klein et al. [11]. The only significant difference reported between the insulins in the latter experiment was a lower within subject variability of the total GIR_AUC_ and GIR_max_ for detemir which we could not reproduce. Our study showed a lower within subject variability for detemir for the time to GIR_max_, but not the other PD outcomes.

Nevertheless, our study confirms the results of these single-injection experiments and suggests a comparable, dose-dependent metabolic activity of detemir and glargine during the first 24 hours in patients with type 2 diabetes and, most importantly, extends these findings to even more severely obese persons. Furthermore, our study design with an extended clamp period allowed to demonstrate a slightly, but significantly enhanced metabolic activity of glargine after 24 hours. In contrast, a much more pronounced difference between the metabolic profiles of detemir and glargine was reported by Lucidi et al who found a 42% higher GIR_AUC_ after glargine in a group of overweight (mean BMI 29 kg/m^2^), insulin-dependent patients with type 2 diabetes [14]. This is the only experiment comparable to ours with regards to its duration, however, major differences are that it used lower insulin doses (0.4 U/kg) and it was performed under steady-state conditions after the basal insulins had been applied for 5 days at bedtime. Despite these important differences their finding of an enhanced late (>16 hours of the clamp) metabolic activity of glargine when compared to detemir agrees with our results. The increased glucose requirements following a single injection of 0.4 U/kg body weight glargine when compared to detemir during the last 4 hours of the 24 hour clamp study of Sørensen et al. in normal weight, non-diabetic subjects adds further support to the notion of an enhanced late metabolic activity of glargine [30].

The finding of an increased metabolic action of glargine (assessed on a unit-per-unit basis) when compared to detemir in this study was also mirrored in a clinical study in subjects with comparable characteristics [31]. The overall clearly more pronounced effect of glargine in this experiment may be explained by the use of the steady-state design, since glargines full metabolic activity may not be obvious after the first injection. This has been suggested in some but not all studies performed in patients with type 1 diabetes [10,32]. Furthermore, the time-point of the insulin injection at bedtime in the study of Lucidi and in the morning in other experiments including ours may be critical due to an interaction of the metabolic effects of the underlying circadian rhythm and the applied insulins [14,29].

Further different properties of the insulins were described in the experiment by Lucidi et al.: free fatty acids were significantly more suppressed with glargine, and increasing obesity attenuated the metabolic activity of detemir but not glargine [14,16]. The enhanced suppression of plasma free fatty acids following glargine in the study by Lucidi et al. is well explained by glargine’s increased metabolic activity in their experiment which we could not reproduce in our clearly more obese patients. Furthermore, we failed to detect any effect of the body mass index on the main pharmacodynamic properties of either insulin. Apart from variations in experimental design the patient characteristics need to be taken into account, and it may well be that with increasing obesity the different characteristics of detemir and glargine become less evident.

Potential limitations of our study need to be addressed. The patients included presented with heterogeneous clinical characteristics. However, the addition of important baseline parameters such as age, sex, BMI, diabetes duration and metabolic control (HbA1c concentrations) as random effects into the statistical model had no significant impact on the pharmacodynamic outcomes. According to our sample size calculation the number of subjects included in the trial was sufficient to detect significant differences between the insulin doses applied but may be insufficient to detect subtle differences between the insulin types. The variability of the results and number of subjects included is well in line with those of comparable studies and the cross-over design chosen allows–at least in part–to overcome this limitation.

The mean insulin dose applied during the high-dose experiments (214 units, 95% CI 174–253) is clearly higher than what is most frequently used in clinical practice, but it is not unheard of. In anyway, the data provide clear evidence that an escalation to very high doses of both insulins may, at least in the short term, be an alternative therapeutic option if other measures to reach glycemic control fail.

## Conclusions

In summary, the present data demonstrate that once daily injections of detemir and glargine provide sustained glucose lowering effects in severely obese patients with type 2 diabetes. Due to its enhanced late metabolic activity beyond 24 hours, the full effect of glargine may not be obvious until a steady-state has been reached after several days. Furthermore, we confirm a clear dose-response relationship of both insulins for the first time in severely obese subjects with a mean BMI of 43 kg/m^2^.

## Supporting information

S1 Consort 2010 checklist (DOC)

S1 Fig Pharmacokinetics. Plasma concentrations (means) of detemir (triangles) and glargine (circles) during the 30 hours clamp period (△ detemir lower dose, ▲ detemir higher dose, ○ glargine lower dose, ● glargine higher dose).
(TIF)

S1 Table Dose (total units) and duration (minutes) of short acting insulin infused to lower and keep blood glucose at 5.5 mmol/l before and immediately after the study drugs were injected at the start of the clamp studies. (DOCX)

S2 Table Within patient variability (CV) of the pharmacodynamic effects of detemir vs. glargine. (DOCX)

S3 Table Mean absolute changes from baseline in plasma FFA, C-peptide and glucagon concentrations during the clamp studies. (DOCX)

S4 Table Area under the curve (AUC) of plasma FFA, C-peptide and glucagon concentrations during the clamp studies. (DOCX)

S1 Dataset (ZIP)

S1 Study protocol (PDF)
