# Supplementary figures and images for: Comparison of the dose-response pharmacodynamic profiles of detemir and glargine in severely obese patients with type 2 diabetes: A single-blind, randomised cross-over trial
Source: PLoS One. 2018 Aug 16;13(8):e0202007. doi: 10.1371/journal.pone.0202007 (PMC6095527; doi:10.1371/journal.pone.0202007)

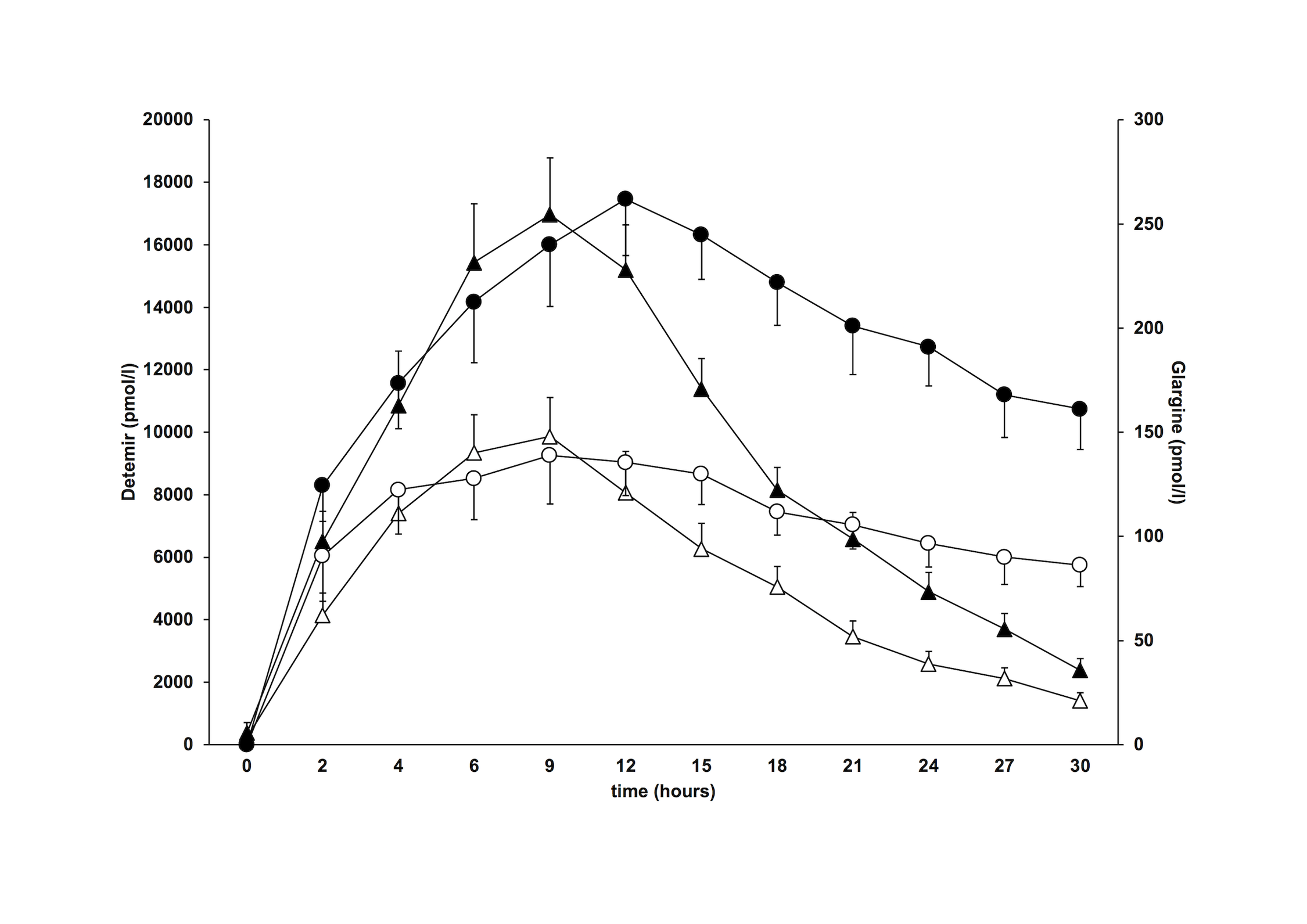

Supplement: S1 Fig — Plasma concentrations (means) of detemir (triangles) and glargine (circles) during the 30 hours clamp period (△ detemir lower dose, ▲ detemir higher dose, ○ glargine lower dose, ● glargine higher dose). (TIF) [file pone.0202007.s002.tif]
