# Supplementary material for: Comparison of the dose-response pharmacodynamic profiles of detemir and glargine in severely obese patients with type 2 diabetes: A single-blind, randomised cross-over trial
Source: PLoS One. 2018 Aug 16;13(8):e0202007. doi: 10.1371/journal.pone.0202007 (PMC6095527; doi:10.1371/journal.pone.0202007)
Supplement: S1 Table — (DOCX) [file pone.0202007.s003.docx]

**S1 Table. Dose (total units) and duration (minutes) of short acting insulin infused to lower and keep blood glucose at 5.5 mmol/l before and immediately after the study drugs were injected at the start of the clamp studies. 0 indicates study drug injection.**

|  | **Detemir** | | **Glargine** | | ***p-values*** | |
| --- | --- | --- | --- | --- | --- | --- |
|  | ***LD*** | ***HD*** | ***LD*** | ***HD*** | ***Insulin type*** | ***Insulin dose*** |
| **Dose to 0 (U)** | 17.8 (0-38.5) | 15.8  (0-42.4) | 17.3  (0-44.2) | 16.8  (0-59.2) | *0.648* | *0.715* |
| **Dose after 0 (U)** | 2.0  (0-27.5) | 2.7  (0-9.9) | 2.5  (0-13.8) | 1.2  (0-12.0) | *0.562* | *0.772* |
| **Time to 0 (min)** | 265  (0-360) | 220  (0-400) | 320  (0-420) | 200  (0-380) | *0.553* | *0.171* |
| **Time after 0 (min)** | 130  (0-410) | 110  (0-270) | 110  (0-390) | 60  (0-290) | *0.502* | *0.542* |

Data are medians and range. LD denotes lower insulin dose, HD denotes higher insulin dose. P-values (Kruskal Wallis tests) are given for differences between insulin type and insulin dose.
