# Supplementary material for: Comparison of the dose-response pharmacodynamic profiles of detemir and glargine in severely obese patients with type 2 diabetes: A single-blind, randomised cross-over trial
Source: PLoS One. 2018 Aug 16;13(8):e0202007. doi: 10.1371/journal.pone.0202007 (PMC6095527; doi:10.1371/journal.pone.0202007)
Supplement: S2 Table — (DOCX) [file pone.0202007.s004.docx]

**S2 Table. Within patient variability (CV) of the pharmacodynamic effects of detemir vs. glargine.**

|  | **Detemir** | **Glargine** | ***p-value*** |
| --- | --- | --- | --- |
| **CV GIR _AUC_** | 80 (52 – 101) | 95 (67 – 117) | *0.309* |
| **CV GIR _max_** | 71 (53 – 85) | 99 (98 – 135) | *0.214* |
| **CV tGIR _max_** | 14 (6 – 19) | 25 (26 – 42) | *0.011* |

Data are % and 95% CI. GIR denotes glucose infusion rate.
