# Supplementary material for: Comparison of the dose-response pharmacodynamic profiles of detemir and glargine in severely obese patients with type 2 diabetes: A single-blind, randomised cross-over trial
Source: PLoS One. 2018 Aug 16;13(8):e0202007. doi: 10.1371/journal.pone.0202007 (PMC6095527; doi:10.1371/journal.pone.0202007)
Supplement: S3 Table — (DOCX) [file pone.0202007.s005.docx]

**S3 Table. Mean absolute changes from baseline in plasma FFA, C-peptide and glucagon concentrations during the clamp studies.**

|  | **Detemir** | | **Glargine** | | ***p-value*** | |
| --- | --- | --- | --- | --- | --- | --- |
|  | ***LD*** | ***HD*** | ***LD*** | ***HD*** | ***Insulin type*** | ***Insulin dose*** |
| **FFA** (μmol/l) | 104  (-25 - 234) | -91  (-262-79) | 79  (-69-228) | -197  (-430-57) | *0.285* | *0.001* |
| **C-peptide** (μg/l) | -0.59  (-1.62-0.44) | -0.71  (-1.27- -0.15) | -0.45  (-1.10-0.21) | -0.98  (-1.40- -0.57) | *0.543* | *0.044* |
| **Glucagon** (ng/l) | 0.22  (-2.02-2.07) | -3.81  (-8.67-1.05) | 0.29  (-8.24-8.80) | -7.05  (-16.32-2.22) | *0.648* | *0.073* |

LD denotes lower insulin dose, HD denotes higher insulin dose. Data are means and 95% CI.
