# Supplementary material for: Comparison of the dose-response pharmacodynamic profiles of detemir and glargine in severely obese patients with type 2 diabetes: A single-blind, randomised cross-over trial
Source: PLoS One. 2018 Aug 16;13(8):e0202007. doi: 10.1371/journal.pone.0202007 (PMC6095527; doi:10.1371/journal.pone.0202007)
Supplement: S4 Table — (DOCX) [file pone.0202007.s006.docx]

**S4 Table. Area under the curve (AUC) of plasma FFA, C-peptide and glucagon concentrations during the clamp studies.**

|  | **Detemir** | | **Glargine** | | ***p-values*** | |
| --- | --- | --- | --- | --- | --- | --- |
|  | ***LD*** | ***HD*** | ***LD*** | ***HD*** | ***Insulin type*** | ***Insulin dose*** |
| **FFA** (μmol/l) | 13797  (11283-16311) | 10101  (7326-12876) | 14971  (11506-18437) | 9702  (6202-13202) | *0.690* | *0.0002* |
| **C-peptide** (μg/l) | 51  (21-81) | 42  (23-60) | 52  (31-74) | 41  (24-59) | *0.933* | *0.008* |
| **Glucagon** (ng/l) | 2019  (1437-2601) | 1775  (1091-2459) | 2132  (1366-2899) | 1844  (1146-2540) | *0.867* | *0.007* |
