# Supplementary material for: Comparison of the dose-response pharmacodynamic profiles of detemir and glargine in severely obese patients with type 2 diabetes: A single-blind, randomised cross-over trial
Source: PLoS One. 2018 Aug 16;13(8):e0202007. doi: 10.1371/journal.pone.0202007 (PMC6095527; doi:10.1371/journal.pone.0202007)
Supplement: S1 Study protocol — (PDF) [file pone.0202007.s008.pdf]

STUDY PROTOCOL:

Pharmacokinetics and pharmacodynamics of insulin detemir (Levemir®) and insulin glargine (Lantus®) after s.c. injection of increasing doses into morbidly obese type 2 diabetic subjects

Jardena Puder, MD, Beat Müller, MD, Stefan Bilz, MD, Ulrich Keller, MD

Division of Endocrinology, Diabetes and Clinical Nutrition,University Hospital Basel, 4031 Basel, Switzerland.

Primary Investigator:
Jardena Puder,
Division of Endocrinology,
Diabetes and Clinical Nutrition
University Hospital Basel,
4031 Basel, Switzerland.
Phone: 061 265 5821
Email: jpuder@uhbs.ch

Date:	Signature:

Table of Contents

1. Rationale 2

2. Objectives: 3

3. Trial design and methods 3
3.1. Trial drugs 3
3.2. Trial population: 3
3.3. Trial schedule: 4
3.4. Potential risks and adverse events 5
3.5. Data analysis and statistics 6

4. Organisation 7
4.1. Specific Location: 7
4.2. Time schedule: 7

5. Informed Consent, Ethical Review, Liability, Regulatory and Economic Considerations 7
5.1. Informed consent 7
5.2. Data collection 7
5.3. Ethical review 8
5.4. Liability 8
5.5. Regulatory considerations 8
5.6. Economic considerations 8
5.7. Sponsoring 8

6. Significance and outlook 8

7. Study investigators 9

9. References: 9

1. Rationale

Interventional trials demonstrated that intensive blood glucose control in patients with type 1 or type 2 diabetes mellitus protects against the onset or progression of long-term micro-vascular complication (1, 2). Insulin therapy is often needed to achieve adequate blood glucose control; it consists injection of a long acting insulin to cover basal needs, and of meal-related short acting insulin. Regarding basal insulin, a preparation with a near peak-less and long-lasting effect that mimics the flat interprandial and basal insulin secretion seen in nondiabetic individuals would be desirable in order to improve glycemic control and to diminish the risk of hypoglycemia (3) .

The human insulin analogues detemir (Levemir®) and glargine (Lantus®) are increasingly used as a basal insulins in type 1 and in type 2 diabetes. Both are long acting insulin analogues indicated for subcutaneous (s.c.) administration. The unique mechanism of protraction of insulin detemir results from a combination of increased self association and albumin binding via the attached C-14 fatty acid, delaying and prolonging absorption (4, 5). Insulin glargine differs from human insulin by replacement of the amino acid asparagine at position A21 by glycine and by the addition of two arginine molecules to the C-terminus of the B-chain (6). After injection into the subcutaneous tissue, the acidic solution of insulin is neutralized, leading to the formation of micro-precipitates from which small amounts if insulin glargine are slowly released (7). The addition of a small amount of zinc (30µg/ml insulin) as a hexamer stabilizing agent further prolongs the duration of action (8).

Previous studies demonstrated both a delayed and a sustained blood glucose-lowering effect with insulin detemir and insulin glargine compared with conventional NPH insulin in healthy subjects (9-11). These results also revealed that a higher molar dose of insulin detemir was needed to achieve comparable glycemic control similar to that observed with NPH insulin (10). Importantly, glucose clamp studies and clinical trials have shown that the variability of the glucose lowering action of insulin detemir and glargine are reduced compared to NPH insulin in diabetic patients. In patients with type 1 diabetes, the mean duration of action of insulin detemir at clinically relevant insulin doses ranged between 12 to 23 hours, while the mean (± SE) duration of action of glargine was 22 ± 4 hours (12, 13). The pharmacokinetics and pharmacodynamics of s.c. injected insulin are dose-dependent. Thus, both the peak and the duration of action were prolonged after injecting increasing doses of regular insulin, rapidly acting insulin analogues or NPH-insulin (14). One study with insulin detemir in patients with type 1 diabetes confirmed a dose-dependent increase in duration of action (12). This is important, as the dosage requirements in type 2 diabetes can be very high, especially in obese subjects with marked insulin resistance. Yki-Järvinen et al reported daily injected doses of up to 168 Units of basal insulin per day (15).

In clinical practice further weight gain with insulin therapy in obese patients with type 2 diabetes is a major barrier to insulin therapy. Interestingly, studies with insulin detemir have shown significantly less weight gain compared to NPH insulin (16). Research concerning the potential mechanisms for this weight-neutral behavior of detemir is ongoing.

In obese patients, doses of up to 1.5-2 U/kg body weight of basal insulin are increasingly used. However, most previous pharmacodynamic studies used lower doses of insulin detemir (0.6 U/kg body weight) (9, 10, 17) or insulin glargine (18-21) and investigated either lean or slightly overweight patients (9, 10, 12, 13, 17-21), but not patients with frank obesity. Thus, pharmacodynamic parameters (maximum action; duration of action) of moderate to high doses of subcutaneously applied detemir and glargine insulin, which are increasingly used in severely obese subjects, have not been reported up to date.

2. Objectives:

The primary aim of this study is to compare the dose-response relationship of moderate and of high doses of insulin detemir and of insulin glargine in 12 severly obese type 2 diabetic subjects.

1. Primary endpoint:
    - Pharmacodynamics:
        - The glucose consumption over time to maintain euglycemia: i.e. the peak of action, the time from peak to half maximal action, the total duration of action will be assessed following a single subcutaneous injection of two different doses of insulin detemir and glargine.
        - The total duration of action is defined as the time between the beginning of action (from the time of 50% reduction in intravenous insulin infusion) until the end of action when plasma glucose consistently exceeds 8.3 mmol/l after the glucose infusion had been stopped, or after 36 h, whichever comes first (12, 13) of both doses and of both insulins.
        - If the glucose-insulin-lowering effects of the 2 insulins are not equal at identical insulin dosages, the pharmacodynamics will be compared at the same effect level (standardized activity ratio, see below).

2. Secondary endpoints:
    - Pharmacokinetics:
        - The plasma peak detemir and glargine levels and the time from the respective peak levels to half maximum levels at the two dosages of both insulins
        - The mean duration of insulin as defined by appropriately elevated detemir and glargine levels at each of the two dosages.
        - The dose-equivalence of both, insulin glargine and detemir in terms of their glucose-lowering effect in obese patients with type 2 diabetes mellitus.
    - Other:
        - The total glucose consumption and the duration of action of the two insulins as a function of the degree of obesity, i.e. of body mass index and central obesity (waist circumference) in obese patients with type 2 diabetes mellitus.

3. Trial design and methods

3.1. Trial drugs

Insulin detemir (Levemir®) will be provided by NovoNordisk Pharma AG, Küsnacht, Switzerland. Insulin glargine (Lantus®, Sanofi-Aventis, Meyrin, Switzerland) will be obtained from the hospital pharmacy, University hospital Basel, Switzerland.

3.2. Trial population:

Twelve morbidly obese subjects with type 2 diabetes (BMI >35 kg/m^2) will be recruited from the outpatient clinic of the Division of Endocrinology, Diabetes and Clinical Nutrition at the University Hospital Basel.

3.2.1. *Inclusion criteria:*

- History of type 2 diabetes mellitus treated with insulin and/or metformin, and/or sulfonylureas
- Age 18-65 years
- Body mass index > 35 kg/m2
- HbA1c >6.0% and < 10%

3.2.2. *Exclusion criteria:*

- Patients treated with thiazolidinediones (glitazones)
- Any significant or active hepatic, cardiovascular, pulmonary, renal, neurological, musculoskeletal, hematological or endocrine disease.
- Pregnant or breast feeding women
- Woman of childbearing potential not using a reliable method of birth control such as oral contraceptives or an IUD.
- Subjects refusing or being unable to give written informed consent

3.3. Trial schedule:

3.3.1. *Screening visit:*

Patients admitted to the outpatient clinic of the Division of Endocrinology, Diabetes and Clinical Nutrition of the University Hospital Basel who are deemed eligible to participate based on medical history and clinical findings will be given a detailed explanation of the study protocol. After obtaining written informed consent a brief physical examination (including, blood pressure, heart rate, body weight and height, waist circumference) will be performed, and a baseline blood sample (including WBC, RBC, creatinine, electrolytes, liver enzymes, lipid profile) will be obtained. A pregnancy test will be obtained in women with childbearing potential.

3.3.2. *Inpatient metabolic studies*

Patients eligible to participate in the study based on the results of the screening visit will be admitted to the Department of Medicine, University Hospital Basel (Petersgraben 4, Klinikum 2) at 7 am after fasting overnight for approximately 40 h, including an overnight stay. In patients on sulfonylureas these will be discontinued for 4 weeks before beginning the study and be withheld during the total duration of the study. Metformin will be paused 48 h (kinetic wash-out) and long-acting basal insulins will be paused 48-72 h prior to the study. If necessary, short-acting insulin will be given up to the evening prior to the study to keep blood glucose levels below 10 mmol/l.

An intravenous cannula will be inserted into an antecubital vein for the infusion of insulin and glucose. A second intravenous canula will be placed in a dorsal hand vein to obtain frequent blood samples for measurement of plasma glucose concentration.

At the beginning of the study a variable intravenous infusion of human soluble insulin will be started at 8 am to reach euglycemia at a plasma glucose level of 5.5 mmol/l within 30-60 minutes. At 10 am a single subcutaneous injection of either insulin detemir or insulin glargine will be administered in the thigh in two different dosages (0.8 and 1.6 IU/kg body weight) in randomized order. Intravenous regular insulin will be tapered when plasma glucose falls below 5.5 mmol/l.

Plasma glucose will be monitored at frequent intervals every 10 to 30 min (goal: plasma glucose 5.5 mmol/l; total blood needed 2 ml/measurement), and glucose 10% will be

administered by continuous i.v. infusion (syringe pump) using an algorithm to maintain plasma glucose levels at 5.5 mmol/l. Blood will also be drawn every 2 h for the first 6 h and subsequently every 6 h for the measurements of the serum concentrations of C-peptide, insulin detemir and insulin glargine using specific radioimmunoassays.

Each subject will be studied at four occasions in order to study the effects of two different doses (0.8 and 1.6 IU/kg body weight) of insulin detemir and of insulin glargine,, respectively. The procedures will be identical during each study. The time period between the different studieis always at least 7 days.

Each inpatient metabolic study will last 36 hours after the injection of insulin detemir or until the plasma glucose will consistently increase to > 8.3 mmol/l in the absence of glucose infusion.

Subjects will not be permitted to eat during the inpatient study, but will be given a dinner meal after the end of the study before leaving the hospital. Noncaloric drinks will be given ad libitum throughout the study procedure. Patients will be blinded regarding the dose and type of insulin.

For each patient, the entire study will be finished after the fourth inpatient study has been completed.

3.4. Potential risks and adverse events

3.4.1. Potential risks:

We consider the risks of this study to be small.

The specific risks are:

Blood Loss: The total blood loss for both parts of the study will be approximately 800 ml. Patients with pre-existing anemia will be excluded and we will consult patients not to donate blood 4-5 months before and after the study. The 4 parts of the study will be spread over 4 months.

Intravenous catheters: Intravenous catheters used during the clamp are associated with a mild to moderate degree of pain upon insertion, and a small risk of localized infection.

Euglycemic clamp: The euglycemic clamp is a safe technique, which has been used by our group for more than 10 years without any side effects. The insulin doses increase plasma insulin levels to concentrations in the range of fasting to low post-prandial concentrations, and will be gradually decreased as soon as the subcutaneously injected insulin detemir will start its effect. The risk of hypoglycemia during the clamp is minimal since 10% glucose is being infused and plasma glucose is monitored every 10-30 min.

3.4.2. Adverse events

The study investigators will oversee and monitor all aspects of the study including subject consent, study conduct, data handling as well as reporting any adverse events to the local ethical comittee and Swissmedic

3.4.3. Assessment of risk:

The procedures included in this study are considered to present only mild risk to research participants. However, the study will be reviewed and reported as required by the “Verordnung über klinische Versuche mit Heilmitteln” des Schweizerischen Bundesrates vom 17. Oktober 2001”.

3.4.4. Attribution of adverse events:

Adverse events will be monitored for every subject participating in the study and attributed to the study procedures/design by the study investigators according to the following categories:

Possible: Adverse event(s) may be related to investigational procedure(s)/agent(s) or other intervention.

Unlikely: Adverse event(s) will doubtfully be related to investigational procedure(s)/agent(s) or other intervention.

3.4.5. Plan for grading adverse events:

The following grade levels will be used in defining the severity of adverse events:

Serious: Death, immediate risk of death, hospitalization or a prolongation of existing hospitalization or a persistent or significant disability/incapacity.

Non-serious: Mild or moderate adverse event or within normal limits.

3.4.6. Plan for reporting serious adverse events:

While no serious adverse events are expected, the study investigators will report any serious adverse events to the local ethical committee and Swissmedic within 48 hours.

3.4.7. Plan for reporting non-serious adverse events:

Non-serious unanticipated adverse events will be reported to the local ethical committee and Swissmedic on an annual basis.

3.4.8. Data and safety review and frequency:

The study investigators will review the data and study conduction on a weekly basis and determine whether the study should continue unchanged or whether modifications to the protocol and/or consent form are required.

3.5. Data analysis and statistics

3.5.1. Null-hypothesis for the primary endpoint:

The total glucose consumption and the mean duration of action will not be different between the two insulins and the two insulin dosages.

3.5.2. Null-hypothesis for the secondary endpoint:

With increasing dosage, the mean duration of action of insulin detemir and insulin glargine will not last over 30 hours

The plasma peak insulin levels and the time from the respective peak levels to half max levels at the two dosages of both insulins will not be different.

The total glucose consumption and the duration of action of the insulins is not correlated to the degree of obesity, i.e. of body mass index and of body composition (central fat accumulation) in obese patients with type 2 diabetes mellitus.

Both insulins (glargine and detemir) are dose-equivalent in terms of their glucose-lowering effect in obese patients with type 2 diabetes mellitus.

3.5.3. Measurements and calculations

Performance of an euglycemic hyperinsulinemic clamp by hourly measurement of plasma glucose, insulin and C-peptide and calculation of the area under the curve (AUC) of the necessary glucose infusion rate (GIR) which is necessary to maintain plasma glucose at 5 mmol/l. For serum insulin and serum C-peptide, descriptive statistics for pharmacokinetic measures are performed (maximal serum insulin concentration [Cmax], time to Cmax [tmax], and AUC; minimal serum C-peptide concentration [Cmin], time to Cmin [tmin] and AUC).

End of insulin action is defined as the time at which plasma glucose consistently increases to > 8.3 mmol/l despite adaptation of glucose infusion.

Since it is debated, that a given unit of insulin detemir is equipotent to a given unit of insulin glargine, we compare the activity ratio (standardized activity ratio) of the two insulins by the total glucose consumption (g glucose/U of insulin administered). This activity ratio will be used to determine further pharmacodynamic effects like the peak of action, the time from peak to half maximal action and the total duration of action.

We will perform a mixed model with a random subject effect in order to calculate differences between the two different dosages and the different insulins (total glucose consumption, duration of action). Among others, covariates that will be assessed include BMI, age, sex.

As there are several primary endpoints, a Bonferroni correction will be applied.

The sample size is small, but was determined by specifying a 95% CI with a relative width of 30% for the duration of action (12). However, as previous studies have used lower total insulin dosages and studied patients for maximal 24 hours, a very meaningful sample size calculation cannot be performed.

1ml of additional serum and plasma will be stored for batch analysis of cytokines and not yet defined parameters (not part of this protocol)

4. Organisation

4.1. Specific Location:

Outpatient Clinic of the Division of Endocrinology, University Hospital Basel and Clinical Research Center, University Hospital Basel.

4.2. Time schedule:

Start of the study: January 2006
Duration: Two years, including recruitment period.

5. Informed Consent, Ethical Review, Liability, Regulatory and Economic Considerations

5.1. Informed consent

Subjects are required to provide written informed consent before enrollment into the study protocol. The consent includes a statement that enrolled subjects agree on the use of their data for scientific purposes. Patients who are not able to give written informed consent are not included in the study. The informed consent document will be used to explain in simple terms the aim of the study, required visits and procedures. The informed consent document contains a statement that the consent is freely given, and that the patient is free to withdraw from the study at any time.

5.2. Data collection

Data collected will be kept confidential and accessible only to researchers involved, the Ethical Committee beider Basel and Swissmedic. Subjects' names will be protected by the use of a unique ID code for each participant. The data records will be stored with the Principal Investigator in a locked room and the electronic data will be kept on a computer with password protection. All study records will be kept indefinitely.

5.3. Ethical review

This protocol will be submitted for approval to the conjoined local Ethical Committee beider Basel and to Swissmedic for approval of the use of insulin detemir (Levemir®, NovoNordisk Pharma AG, 8700 Küsnacht, Schweiz) and glargine (Lantus®, Sanofi-Synthélabo Schweiz AG, 1217 Meyrin, Schweiz).

5.4. Liability

The study procedures will be covered by the general liability insurance RIMAS* of the University hospital Basel, as will be certified by Jürg Müller (Rechtsdienst, University Hospital Basel).

(* RIMAS, Insurance-Broker AG, Hanspeter Kolmos, Fischmarkt 10, Postfach 1919, CH-4001 Basel, Tel.: 061 269 81 13, Fax: 061 269 81 10).

5.5. Regulatory considerations

This study will be conducted in accordance with the ethical principles stated in the most recent version of the Declaration of Helsinki or the applicable International Conference on Harmonization (ICH) guidelines on good clinical practice, whichever represents the greater protection of the individual.

The study will not be started until approval of the study protocol by the local ethical committee and by Swissmedic.

Data collected will be kept confidential and accessible only to researchers involved, the ethical review committee and Swissmedic. The data records will be stored in a locked room and the electronic data will be kept on a computer with password protection. All study records will be kept for at least 15 years.

5.6. Economic considerations

No additional costs will arise for patients through participation in the study. There will be no charges for any of the testing procedures including blood measurement, the euglycemic clamp study, the overnight stay and the provided dinner. Since participation does not provide any direct medical benefit to the patients, they will be reimbursed with CHF 1500.- for the participation in the four parts of the study (Insulin detemir and insulin glargine at the 2 dose levels). The costs of the metabolic studies will be covered by the Primary Investigator.

5.7. Sponsoring

This is an investigator-driven study. The primary investigator is the sponsor. The study will be supported by an unrestricted grant of Novo Nordisk Pharmaceutical Company Küsnacht, Switzerland.

6. Significance and outlook

With the increasing incidence and prevalence of type 2 diabetes mellitus, insulin therapy will be of major importance, because insulin is highly effective. Long-acting insulins like insulin detemir and insulin glargine are being used more and more frequently. Safety data about high doses of insulin are necessary and reassuring for physicians treating obese patients with insulin. As all insulins show a dose-dependent prolongation of their action profile, an examination of the effect of high doses, as often needed in insulin resistant states, is important for the prevention of hyper- and hypoglycaemia and thus for the safety and efficacy of insulin therapy.

7. Study investigators

Name	Institution	Phone	Email
**Jardena Puder, MD**	Endocrinology, Diabetes and Clinical Nutrition, University Hospital Basel, Switzerland	061 265 5821	jpuder@uhbs.ch
**Prof. Ulrich Keller, MD**	Endocrinology, Diabetes and Clinical Nutrition, University Hospital Basel, Switzerland	061 265 5073	ukeller@uhbs.ch
**Stefan Bilz, MD**	Endocrinology, Diabetes and Clinical Nutrition, University Hospital Basel, Switzerland	061 265 5068	bilzs@uhbs.ch
**Prof. Beat Müller, MD**	Endocrinology, Diabetes and Clinical Nutrition, University Hospital Basel, Switzerland	061 265 5079	happymiller@bigfoot.com

9. References:

1. 1993 The effect of intensive treatment of diabetes on the development and progression of long-term complications in insulin-dependent diabetes mellitus. The Diabetes Control and Complications Trial Research Group. N Engl J Med 329:977-86
2. 1998 Intensive blood-glucose control with sulphonylureas or insulin compared with conventional treatment and risk of complications in patients with type 2 diabetes (UKPDS 33). UK Prospective Diabetes Study (UKPDS) Group. Lancet 352:837-53
3. **Zinman B** 1989 The physiologic replacement of insulin. An elusive goal. N Engl J Med 321:363-70
4. **Kurtzhals P, Havelund S, Jonassen I, Kiehr B, Larsen UD, Ribel U, Markussen J** 1995 Albumin binding of insulins acylated with fatty acids: characterization of the ligand-protein interaction and correlation between binding affinity and timing of the insulin effect in vivo. Biochem J 312 (Pt 3):725-31
5. **Whittingham JL, Havelund S, Jonassen I** 1997 Crystal structure of a prolonged-acting insulin with albumin-binding properties. Biochemistry 36:2826-31
6. **Hirsch IB** 2005 Insulin analogues. N Engl J Med 352:174-83
7. **Bolli GB, Di Marchi RD, Park GD, Pramming S, Koivisto VA** 1999 Insulin analogues and their potential in the management of diabetes mellitus. Diabetologia 42:1151-67
8. **Pieber TR, Eugene-Jolchine I, Derobert E** 2000 Efficacy and safety of HOE 901 versus NPH insulin in patients with type 1 diabetes. The European Study Group of HOE 901 in type 1 diabetes. Diabetes Care 23:157-62
9. **Brunner GA, Sendhofer G, Wutte A, Ellmerer M, Sogaard B, Siebenhofer A, Hirschberger S, Krejs GJ, Pieber TR** 2000 Pharmacokinetic and pharmacodynamic properties of long-acting insulin analogue NN304 in comparison to NPH insulin in humans. Exp Clin Endocrinol Diabetes 108:100-5
10. **Heinemann L, Sinha K, Weyer C, Loftager M, Hirschberger S, Heise T** 1999 Time-action profile of the soluble, fatty acid acylated, long-acting insulin analogue NN304. Diabet Med 16:332-8
11. **Wang F, Carabino JM, Vergara CM** 2003 Insulin glargine: a systematic review of a long-acting insulin analogue. Clin Ther 25:1541-77, discussion 1539-40
12. **Plank J, Bodenlenz M, Sinner F, Magnes C, Gorzer E, Regittnig W, Endahl LA, Draeger E, Zdravkovic M, Pieber TR** 2005 A double-blind, randomized, dose-response study investigating the pharmacodynamic and pharmacokinetic properties of the long-acting insulin analog detemir. Diabetes Care 28:1107-12

13. **Lepore M, Pampanelli S, Fanelli C, Porcellati F, Bartocci L, Di Vincenzo A, Cordoni C, Costa E, Brunetti P, Bolli GB** 2000 Pharmacokinetics and pharmacodynamics of subcutaneous injection of long-acting human insulin analog glargine, NPH insulin, and ultralente human insulin and continuous subcutaneous infusion of insulin lispro. Diabetes 49:2142-8
14. **Pickkup JC WG** 2003 Textbook of Diabetes, 3 ed. Blackwell Science
15. **Yki-Jarvinen H, Ryysy L, Nikkila K, Tulokas T, Vanamo R, Heikkila M** 1999 Comparison of bedtime insulin regimens in patients with type 2 diabetes mellitus. A randomized, controlled trial. Ann Intern Med 130:389-96
16. **Fritsche A, Haring H** 2004 At last, a weight neutral insulin? Int J Obes Relat Metab Disord 28 Suppl 2:S41-6
17. **Heise T, Nosek L, Ronn BB, Endahl L, Heinemann L, Kapitza C, Draeger E** 2004 Lower within-subject variability of insulin detemir in comparison to NPH insulin and insulin glargine in people with type 1 diabetes. Diabetes 53:1614-20
18. **Owens DR, Coates PA, Luzio SD, Tinbergen JP, Kurzhals R** 2000 Pharmacokinetics of 125I-labeled insulin glargine (HOE 901) in healthy men: comparison with NPH insulin and the influence of different subcutaneous injection sites. Diabetes Care 23:813-9
19. **Heinemann L, Linkeschova R, Rave K, Hompesch B, Sedlak M, Heise T** 2000 Time-action profile of the long-acting insulin analog insulin glargine (HOE901) in comparison with those of NPH insulin and placebo. Diabetes Care 23:644-9
20. **Luzio SD, Beck P, Owens DR** 2003 Comparison of the subcutaneous absorption of insulin glargine (Lantus) and NPH insulin in patients with Type 2 diabetes. Horm Metab Res 35:434-8
21. **Rave K, Nosek L, Heinemann L, Frick A, Becker R** 2003 Time-action profile of the long-acting insulin analogue insulin glargine in comparison to NPH insulin in Japanese volunteers. Diabetes Metab 29:430-1
